# Editorial: Antiviral options for emerging and reemerging viral diseases: current therapeutics, novel drug candidates and new approaches

**DOI:** 10.3389/fcimb.2024.1497018

**Published:** 2024-10-17

**Authors:** Clement Meseko, Melvin Sanicas, Yash Gupta, Binod Kumar

**Affiliations:** ^1^ Regional Centre for Animal Influenza, National Veterinary Research Institute, Vom, Nigeria; ^2^ Medical and Clinical Development, Clover Biopharmaceuticals, Boston, MA, United States; ^3^ Department of Medicine, Penn State Health Milton S. Hershey Medical Center, Hershey, PA, United States; ^4^ Department of Antiviral Research, Institute of Advanced Virology, Thiruvananthapuram, Kerala, India

**Keywords:** coronavirus, SARS-CoV2, COVID-19, influenza virus, herpesvirus, cellular signaling, antiviral drugs, vaccines

Emerging and re-emerging viral infections, which cause millions of deaths worldwide, pose a constant threat to both human health and the global economy. The world has witnessed several epidemics and pandemics caused by viral infections, underscoring the urgent need for both established and novel antiviral drugs. Viral infections have the potential to spread rapidly, leading to high rates of morbidity and mortality. This is often due to either insufficient immunity in the population or a lack of effective treatment options.

The topics covered in this Research Topic highlight the antiviral options for emerging and reemerging viral infections. The recent SARS-CoV-2 pandemic accelerated the search for novel antiviral treatment options for COVID-19, a viral disease that demonstrated the zoonotic potential of the virus. The review article by Marwaha discussed the SARS-CoV2-associated long-term COVID-19 (Long COVID) which impacted several million patients worldwide. The author highlighted the dual role of Tau protein: in its normal physiological state, it serves as a protective agent against DNA damage and regulates DNA packaging, while in its pathogenic state, it induces oxidative stress, disrupts the nucleoskeleton, and triggers apoptotic cell death. The author conducted a literature search that linked the tau protein to COVID-19. It was observed that the tissue samples of the brain from autopsies of COVID-19 patients had tau deposits. A similar observation was made in the organoid model of SARS-CoV2 infection, which called for further research on tau phosphorylation. The author suggested the possibility of using Tau protein in CSF as a potential biomarker for long-term COVID.

Similarly, influenza viruses have long been linked to frequent epidemics and occasional pandemics, and numerous studies have been published exploring various antiviral strategies to manage these infections (Kumar et al., 2018; Asha et al., 2019, 2023). Viruses often hijack host cellular machinery and utilize host cellular signaling pathways during their replication cycle (Lekshmi et al., 2023). The research article by Hoffmann et al. also demonstrated that two crucial viral pathogens, influenza viruses and SARS-CoV-2, rely on the Raf/MEK/ERK signaling pathway for their replication. This was demonstrated when a small-molecule MEK inhibitor zapnometinib, was tested side by side against both influenza A virus (IAV) and SARS-CoV2 to test its antiviral efficacy. The study found that influenza A virus (IAV) exhibits a greater dependence on the active Raf/MEK/ERK pathway compared to SARS-CoV-2, making IAV more susceptible to treatment with zapnometinib. The study highlighted the promising role of zapnometinib in managing both SARS-CoV2 and IAV infections in a recent and an upcoming phase II clinical trial respectively.

Another article by Meseko et al. reviewed the antiviral options and therapeutics against Influenza. The authors reviewed the need for new approaches to influenza chemotherapeutics to mitigate the severe complications and mortality caused by the continuously emerging novel strains of influenza viruses. The review article provides an extensive review of the history and latest developments made in the field of influenza anti-infectives and other antiviral strategies citing several research studies that focused on the assessment of various antiviral compounds against influenza. The authors discussed articles that showed antiviral options against influenza by targeting either the viral components or the host cellular factors. Finally, the authors also highlighted the clinical trials and outcomes of influenza therapeutics.

In another review, Szabo et al. addressed the challenges of managing SARS-CoV-2 infections, which are exacerbated by the ongoing emergence of new SARS-CoV-2 variants. The authors suggested that a promising antiviral approach is to target and block viral entry into host cells. The article emphasized the role of natural products as coronavirus inhibitors and revealed several unique advantages of utilizing natural products due to their diverse bioactivity and ability to work synergistically with other drugs. The authors summarized the recent reports on coronavirus entry inhibitors derived from plants, honey, and marine sponges.

Viruses with zoonotic potential have always been a threat to human health (Asha and Kumar, 2019). In an interesting research study, Ashley et al. demonstrated the utilization of an *in silico* approach to target the A42R protein of the monkeypox virus (Mpox) to inhibit its infection in target cells. The authors noted that the A42R profilin-like protein of Mpox, which plays a key role in cell development and motility, may be a critical target for drug development. They used computational approaches to screen a library of 36,366 compounds from the Traditional Chinese Medicine (TCM), AfroDb, and PubChem databases and selected seven compounds with a higher likelihood of binding to the A42R protein of the virus thus suggesting the potential of these compounds to be developed against Mpox. Since this protein is highly conserved among orthopoxviruses, the A42R inhibitors may be widely used to also target other members of the same family.

In addition to viruses that infect humans, there are several that specifically target birds, animals, and aquatic wildlife, posing significant threats to the food industry and animal farming (Meseko et al., 2023a). An article by Chen et al. similarly explained how viral infections could pose a significant threat to the equine industry. The authors conducted both *in vitro* and *in vivo* investigations to analyze the effectiveness of Rutin, a flavonoid with multiple pharmacological properties, against equine herpesvirus infection. They showed that Rutin exhibited an inhibitory effect against equine herpesvirus-8 at multiple stages of the viral life cycle. The experiments done on the mouse model revealed that administration of Rutin resulted in a substantial reduction of viral titer in the lungs of mice by targeting the Nrf2/HO-1 signaling pathway-mediated antioxidant response. The study underscores the therapeutic potential of Rutin in controlling the equine herpesvirus-8 infections.

Although significant progress has been made in the field of antiviral drug discovery, vaccination is still considered the gold standard. With the emergence of SARS-CoV2 and COVID-19 disease, a race for the development of a vaccine began. This also led to research focusing on various vaccine strategies against SDARS-CoV2. The article by Hao et al. reviewed the advancements of virus-like particle-based vaccines against SARS-CoV2. The authors summarized the advantages of the VLP platform, strategies for antigen display, and the current progress of clinical trials in this field. The authors emphasized that VLP-based vaccines have the potential to prevent future coronavirus pandemics.

Overall, this Research Topic highlights different strategies for managing viral infections. The articles emphasize the significance of both emerging and re-emerging viral threats and explore the evaluation of antiviral drugs, vaccine strategies, and alternative approaches to control viral diseases.
